# Targeting rare splicing defects: Antisense oligonucleotides offer a therapeutic strategy in FRDA

**DOI:** 10.1016/j.omtn.2025.102723

**Published:** 2025-10-13

**Authors:** Laurie M.C. Kerkhof, Ronald A.M. Buijsen

**Affiliations:** 1 Department of Human Genetics, Leiden University Medical Center, Leiden, the Netherlands; 2 Dutch Center for RNA Therapeutics, Leiden University Medical Center, Leiden, the Netherlands

## Main text

Friedreich’s ataxia (FRDA) is an autosomal recessive, multisystem disorder caused by mutations in the *FXN* gene, progressively affecting the nervous system, heart, and other organs.^1^ A recent study published in *Molecular Therapy Nucleic Acids* by Yameogo et al*.* describes a novel, intronic point mutation that disrupts canonical splicing and severely reduces *FXN* mRNA and frataxin protein levels.^2^ Using patient-derived cells, the authors demonstrated that antisense oligonucleotides (ASOs) targeting splicing regulatory elements effectively restore splicing deficits and increase frataxin expression. While encouraging in cell-based studies, this strategy is limited to a small subset of individuals with FRDA carrying these rare mutations and requires functional validation in disease-relevant tissues.

### Identification of a novel *FXN* point mutation affecting splicing

**Figure 1:**
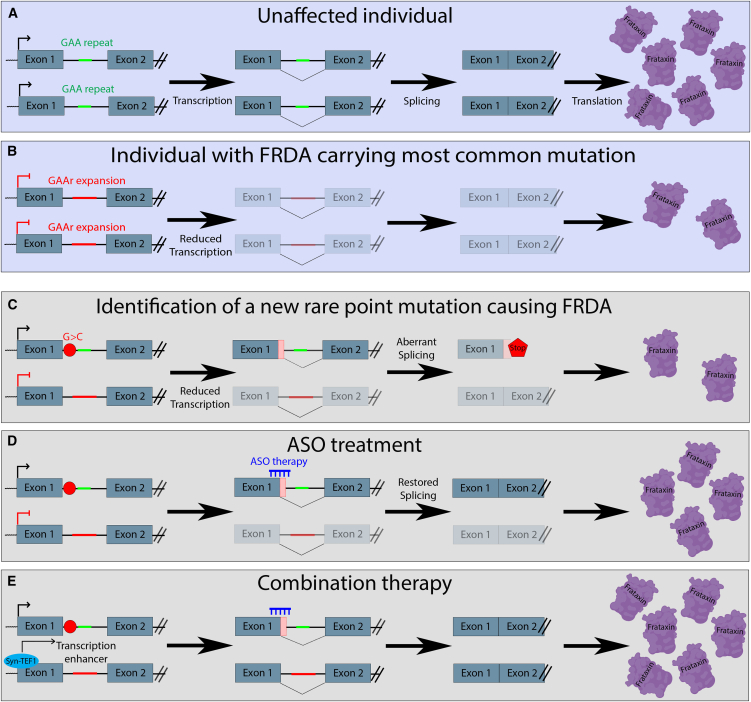
Overview of the genetic mutations, splicing defects, *FXN* mRNA, and frataxin protein levels observed in FRDA (A) Situation in an unaffected individual leading to normal transcription, splicing, and protein translation. (B) Situation in an individual with FRDA carrying the most common mutation, a bi-allelic GAA repeat expansion leading to reduced transcription, reduced *FXN* mRNA and frataxin protein. (C) Identification of a new point mutation on one allele, accompanied by a GAA repeat expansion on the other allele, leading to reduced transcription, aberrant splicing, and less frataxin protein produced. (D) ASO therapy targeting the point mutation resulting in splicing correction and partial restoration of *FXN* mRNA and frataxin protein levels. (E) Combined treatment of the ASO targeting the point mutation and Syn-TEF1, leading to a cumulative enhanced effect restoring transcription, aberrant splicing, and *FXN* mRNA and frataxin protein levels. Created with the help of Biorender.

FRDA is most commonly caused by GAA repeat expansions in intron 1 of the *FXN* gene, which lead to reduced production of the mitochondrial protein frataxin (Figures 1A and 1B).^3^ In a minority of patients, one allele carries this GAA repeat expansion, while the other harbors a point mutation that abolishes functional frataxin production.^4^ Yameogo et al. identified such a point mutation in intron 1 of the *FXN* gene, accompanied by an expanded GAA repeat on the other allele. This novel point mutation induces alternative splicing that most notably introduces a premature stop codon, leading to reduced *FXN* mRNA and frataxin protein levels in patient-derived fibroblasts (Figure 1C).^2^

This finding broadens the mutational spectrum of *FXN* and underscores the importance of investigating non-coding variants as contributors to FRDA. Particularly in individuals who remain undiagnosed after standard testing, comprehensive genetic analyses that include intronic and regulatory regions are essential. More broadly, many genetic disorders remain unresolved until non-coding variants are considered, highlighting the need for extensive diagnostic approaches across diseases. Because splicing can differ across tissues and cell types, testing of splicing in blood or fibroblasts may not reveal all pathogenic variants. In such cases, transcriptomic analyses should be performed in disease-relevant tissues or cells. Patient-derived induced pluripotent stem cells (iPSCs) offer a valuable alternative, as they can be generated by reprogramming skin, blood, or urine cells into a pluripotent state and then differentiated into virtually any cell type for disease-relevant studies.

### Targeted ASO treatment repairs alternative splicing and increases frataxin levels

Omaveloxolone (Skyclarys) is the only approved therapy for FRDA, although many other therapeutic strategies are currently being evaluated in clinical trials.^5^^,^^6^ Omaveloxolone works by boosting the transcription factor Nrf2, which increases the expression of cytoprotective proteins. This helps the cell to resist oxidative stress and prevents subsequent damage. However, it does not correct the underlying frataxin deficiency. To address this root problem, ASOs are a promising approach, and several splice-modulating ASOs for other disorders are already approved by the Food and Drug Administration and European Medicines Agency.^7^ These small, lab-made molecules can be designed to specifically target genetic errors and aid cells in producing the missing protein. Yameogo et al. tested multiple ASOs in patient-derived fibroblasts. Their lead ASO, ASO-82, increased *FXN* mRNA and frataxin protein to about 50% of levels observed in unaffected controls (Figure 1D). To evaluate the effect of ASO-82 on aberrant splicing, minigene constructs carrying the point mutation were transfected into HEK293T cells. Treatment of these cells with ASO-82 restored normal splicing, leading to correction at both the RNA and protein level.^2^ To further increase frataxin levels, repeated delivery of ASO-82 was tested. This resulted in a significant increase in *FXN* transcripts compared to single dosing, showing that increased availability of ASO-82 can further upregulate *FXN* expression.

Despite these promising results, the study was limited to fibroblasts and minigene assays, without functional or extensive toxicity testing. Generating animal models to study these rare mutations is often not feasible, making patient-derived iPSCs particularly valuable. Future studies could rapidly obtain iPSCs from patient blood or skin to generate cardiac or brain organoids, enabling the assessment of functional rescue and cell viability in the tissues most affected by FRDA.^8^ Although this approach will only benefit a small subset of patients, it offers hope for patients with FRDA with rare point mutations causing aberrant splicing. Beyond FRDA, these findings offer promise for individuals affected by other rare, mutation-specific genetic conditions. Drug development for rare diseases is challenging due to small patient populations, regulatory hurdles, and high costs. Nevertheless, collaborations between academia and industry, such as the N1C Collaborative and n-Lorem, are fostering the development of personalized, mutation-specific therapies. Equally important is the open sharing of data and resources, which can accelerate progress and maximize the impact of these efforts.

### Combination therapy cumulatively increases frataxin levels

Syn-TEF1, also known as DT-216, is an experimental therapy for FRDA that also aims to fix the root cause of the disease. Syn-TEF1 works like a molecular helper that allows the cell’s machinery to read through the GAA repeat expansion and produce normal amounts of *FXN* mRNA and frataxin protein. Early human studies have shown that syn-TEF1 can increase frataxin levels in muscles (NCT05573698). Simultaneously targeting splicing deficits in one allele with ASO-82 and transcription deficiency in the other allele with Syn-TEF1 significantly increased *FXN* mRNA levels and frataxin protein levels compared to untreated cells or cells receiving only one treatment. This demonstrates a cumulative effect of the combination therapy (Figure 1E).^2^

The observed cumulative effect of combination therapy opens the door for using similar combination therapies in FRDA and other very rare genetic disorders. However, before combination therapies can be tested in patients, various obstacles first need to be overcome. Despite current efforts for improvement, systemic delivery of ASOs has proven to be quite challenging with limited ASO stability and distribution to target tissues.^7^ Combinatorial therapy will further complicate delivery as this might warrant different delivery strategies for each individual therapy. Furthermore, pharmacokinetics of the individual drugs and the pharmacological interrelation between the different drugs should be well understood to ensure correct dosing and avoid adverse events or toxicity.^9^

### Conclusion

This study by Yameogo et al*.* highlights the potential of ASOs to correct rare splicing defects in *FXN* and restore frataxin expression in FRDA patient cells. While highly effective *in vitro*, the therapy is currently applicable only to a small subset of patients with specific mutations, and functional testing in relevant tissues remains necessary. The combination of ASOs with transcription activation strategies demonstrates that cumulative or synergistic treatments offer a promising avenue to enhance therapeutic outcomes. Overall, these findings underscore the importance of personalized, mutation-specific approaches and the need for continued research and collaborations to translate these strategies into clinically effective therapies.

## Declaration of interests

R.A.M.B. is a member of the Scientific Advisory Council of the Oligonucleotide Therapeutics Society and chair of the Stichting Dutch Antisense Therapeutics.
